# Inequalities in multimorbidity in South Africa

**DOI:** 10.1186/1475-9276-12-64

**Published:** 2013-08-20

**Authors:** John Ele-Ojo Ataguba

**Affiliations:** 1 Health Economics Unit, School of Public Health and Family Medicine, University of Cape Town, Observatory, Cape Town 925, South Africa

**Keywords:** Multimorbidity, Socioeconomic inequality, South Africa

## Abstract

**Background:**

Very little is known about socioeconomic related inequalities in multimorbidity, especially in developing countries. Traditionally, studies on health inequalities have mainly focused on a single disease condition or different conditions in isolation. This paper examines socioeconomic inequality in multimorbidity in illness and disability in South Africa between 2005 and 2008.

**Methods:**

Data were drawn from the 2005, 2006, 2007, and 2008 rounds of the nationally representative annual South African General Household Surveys (GHS). Indirectly standardised concentration indices were used to assess socioeconomic inequality. A proxy index of socioeconomic status was constructed, for each year, using a selected set of variables that are available in all the GHS rounds. Multimorbidity in illness and disability were constructed using data on nine illnesses and six disabilities contained in the GHS.

**Results:**

Multimorbidity affects a substantial number of South Africans. Most often, based on the nine illness conditions and six disability conditions considered, multimorbidity in illness and multimorbidity in disability are each found to involve only two conditions. In 2008 in South Africa, the multimorbidity that affected the greatest number of individuals (0.6% of the population) combined high blood pressure (BP) with at least one other illness. The combination of sexually transmitted diseases (STDs) and other condition or conditions is the least reported (i.e. 0.02% of the population). Between 2005 and 2008, multimorbidity in illness and disability is more prevalent among the poor; in disabilities this is yet more consistent. The concentration index of multiple illnesses in 2005 and 2008 are −0.0009 and −0.0006 respectively. The corresponding values for multiple disabilities are −0.0006 and −0.0006 respectively.

**Conclusion:**

While there is a dearth of information on the socioeconomic distribution of multimorbidity in many developing countries, this paper has shown that its distribution in South Africa indicates that the poor bear a greater burden of multimorbidity. This is more so for disability than for illness. This paper argues that, given the high burden and skewed socioeconomic distribution of multimorbidity, there is a need to design policies to address this situation. Further, there is a need to design surveys that specifically assess multimorbidity.

## Introduction

Recently, there has been a renewed research interest in multimorbidity (i.e., the case where an individual suffers from two or more disease conditions at the same time) [1,2]. However, this research area remains in its infancy [2]. Though its epidemiological pattern is similar to that of non-communicable diseases [3], very little is known about the socioeconomic distribution of multimorbidity. Is the usual reported socioeconomic gradient for ill-health also present for multimorbidity? Traditionally, studies on health inequalities have focused mainly on a single disease condition or different disease conditions in isolation [4-6] but not multimorbidity *per se*. The dearth of studies relevant to this issue, even in industrialised countries, is surprising given that prevalence of multimorbidity is very high in these countries. Indeed, multimorbidity in industrialised countries is described as the rule rather than the exception, at least in primary care [2]. For instance in the United States, it is estimated that over 80 million people will be affected by multimorbidity by 2020 [2].

The prevalence of multimorbidity is generally associated with “increasing age, lower level of education, and public health insurance” [1] (p.367). Thus, its burden increases as a population ages [7]. This can have considerable impact on families, especially in settings where poverty is widespread and comprehensive health care is lacking [7]. However, in resource-poor countries, including those in Africa, research into inequalities in multimorbidity is even more limited. It is arguably true that, proportionately, the current burden of multimorbidity in these settings far outstrips that of developed and industrialised countries [8]. Multimorbidity is also noted to reduce quality of life significantly. This is “not only in terms of how people felt about their lives generally, but also in terms of the extent of their psychological distress” [9] (p.202). This will further increase the cost of treating multimorbidity. For instance in Australia, it was found that the cost of treating a person with multimorbidity is five times that for other people with only one illness [9].

In South Africa, inequalities in health have been extensively reported with the poor bearing a disproportionate burden [4,10]. As noted earlier, such studies did not focus on multimorbidity but on either a specific group (e.g., children) or different disease conditions. In order to provide more pertinent empirical evidence, this paper explores inequality in multimorbidity generally in South Africa. Specifically, the paper examines socioeconomic inequality in multimorbidity in *illness* and *disability* between 2005 and 2008.

## Methodology

### Data

Data for analysis were drawn from the nationally representative annual South African General Household Surveys (GHS) conducted by Statistics South Africa – the national statistical authority. The GHS have been carried out by Statistics South Africa on an annual basis since 2002. They were designed to assess multiple facets of the living conditions and wellbeing of South African households including quality of service delivery in key service sectors of the economy. The surveys cover six broad areas including education, health and household access to services and facilities [11]. The GHS use a multi-stage stratified sampling design with probability-proportional-to-size. The first level of stratification is the province and the second-tier stratification is district councils. The 2005, 2006, 2007 and 2008 rounds were selected with respective sample sizes as 28 129, 28 002, 29 311 and 24 293 households [11-14]. Recently, the GHS dataset has been used to examine unemployment issues [15] and socioeconomic health inequality [4] in South Africa.

The GHS questionnaire does not directly ask specific questions about multimorbidity. This is a common feature with many national general household surveys. From the literature, multimorbidity can be expressed either by counting the number of morbidities or by constructing an index of multimorbidity. The index of multimorbidity has the advantage of accounting for both the number and severity of diseases. However its data requirements limit its applicability [9]. This paper, therefore, uses the count approach by considering multiple illnesses or disabilities that occur within the recall period to construct indicators of multimorbidity [16]. Using this approach, indicators were obtained for multimorbidity in illness and multimorbidity in disability. While this assumption for illness is debatable, it is less so for multimorbidity in disability. This is because disabilities are generally long-term conditions [17] which mean they are most likely to be concurrent conditions.

Based on Valderas *et al.*[16], three categories/indicators of multimorbidity were created for each of the illness and disability group (i.e., for co-occurrence). Each category was constructed as a dichotomous variable indicating multimorbidity. The first category for illnesses considers having an illness (e.g. depression) in addition to one other illness. The second category for illnesses considers having an illness in addition to two other illnesses. The third category for illnesses covers having an illness in addition to at least three or more illnesses. Similar categorisations were done for the disabilities group. Due to the cumbersome nature of the categorisations, illnesses and disabilities were not combined. In relation to co-occurrence including synchronous occurrence, the recall periods for the GHS were used as multimorbidity can be assessed for “disorders co-occurring across a period of time but not necessarily at the same time” [16] (p.358). None of these conditions however was considered as the primary or index condition [16]. The inability of this paper to make such distinction was based on the way the data were captured.

For illnesses, based on the data from GHS, the following types were considered: diarrhoea, trauma, tuberculosis (TB), drug and substance abuse (drugs), depression, diabetes, high/low blood pressure (BP), human immunodeficiency virus (HIV), and sexually transmitted diseases (STDs). Disabilities included sight, hearing, and speech, physical, intellectual, and emotional disability. These conditions were self-reported by individuals from a list presented to them (see Additional file 1). The recall period is one month for illnesses and six months as a minimum for the condition to be considered a disability. All the listed conditions were considered with the exception of flu/acute respiratory tract infection because flu, a common condition, as opposed to acute respiratory tract infection is most frequently reported. The paper uses the individual as the unit of analysis.

### Statistical methods

This paper uses concentration indices that have been extensively used to analyse inequalities in health. They are used to assess relative inequality in health. Compared to other measures of inequality, concentration indices yield consistent ranking of units across socioeconomic groupings; they are sensitive to changes in population distribution across socioeconomic groups and are consistent with experience of health (or ill-health) across the distribution of socioeconomic status (SES) [18,19]. This index is also often further standardised, for instance, to account for age-sex variations in reported health or health outcomes. This standardisation is used to describe the distribution of health/ill-health by socioeconomic groups conditional on confounding demographic factors such as age and sex. In this paper, indirect standardisation was used to correct the distribution of reported health/ill-health by comparing it with that expected of the actual age/sex distribution [19,20].

For an ill-health variable (*h*) (which in this case is any of the dichotomous variables indicating multimorbidity) with mean (*μ*) and the rank of the SES measure (*r*), the indirectly standardised concentration index (*β*) is obtained as an estimate from the simple ordinary least squares (OLS) regression.

(1) $$2\sigma_{r}^{2}\left(\frac{h_{i}}{\mu }\right)=\alpha +\beta_{1}r_{i}+\underset{j}{\sum }\varphi_{j}x_{\mathrm{ij}}+\epsilon_{i}$$

where *x*_*ij*_ are the confounding variables (age and sex in this case), $\sigma_{r}^{2}$ is the variance of the rank of the SES measure and *ϵ* is the stochastic error term [19,20]. The concentration index (*β*) measures the extent of inequalities in health (ill-health) that are systematically associated with socioeconomic status [18].

The value of the concentration index lies between −1 (i.e., when all the population’s ill-health is concentrated on the most disadvantaged person) and +1 (when all the population’s ill-health is concentrated on the least disadvantaged person). A concentration index value of zero indicates either that the population’s ill-health is evenly concentrated along the distribution of SES or that on average, positive and negative effects cancel out across the SES distribution [4]. In general, a positive concentration index indicates that the distribution of ill-health is higher among the richer SES groups while a negative index indicates the opposite.

With dichotomous variables, the concentration index will not lie within the normal bands but between *μ* -1 and 1- *μ* for large samples [21,22]. This suggests the need for some form of normalisation. This paper uses Erreygers’ normalisation procedure. Wagstaff [22] has shown that Erreygers’ [23] index (or correction of the concentration index) can be conveniently written as:

(2) $$E_{c}=\left(4\mu /b-a\right)\cdot C$$

where *C* = the standard concentration index, *μ* is the mean of the health/ill-health variable with its range defined as (*b* – *a*).

### Measuring SES

Debates exist as to the right measure of SES for inequality analysis [24,25]. While SES can be measured using income, expenditure, education, class, or a composite index, this paper uses composite indices as proxy of socioeconomic status [26] based on selected variables (as set out below). This is because the datasets do not contain reliable information on household income and expenditure. The procedure of principal components analysis was used to compute the composite indices [26]. Because the paper uses several rounds of the GHS, the same set of eleven variables (type of dwelling, roof, and wall material, access to safe drinking water, toilet, and source of energy for lighting, and ownership of car, landline, cell phone, TV, and radio) were selected and used to construct the index in each year. Dummy variables were created for each variable signifying the presence of the item in question. Basically, principal components analysis uses statistical techniques to determine the weights (*w*_*k*_ as shown below) attached to each variable in aggregating them into an index.

The composite index value for individual *i* (*IC*_*i*_) is computed as:

(3) $$IC_{i}=\overset{11}{\underset{k=1}{\sum }}\left(\frac{\left(a_{\mathrm{ik}}-{\bar{a}}_{k}\right)}{s_{k}}w_{k}\right)$$

where *a*_*ik*_ is the value of the variable (dummy) *k* for household *i*, *a*_*k*_ is its sample mean, *s*_*k*_ is its sample standard deviation, and *w*_*k*_ are the weights obtained from the first principal component.

These composite indices were used to rank the sample from poorest to richest. Stata® version 12 [27] was used for all analyses.

## Results

Over 50% of the population is female with an average age of 27 years (Table 1). The majority of the population is single/never married with only a few (<2%) divorced/separated. Only a few people (<3%) had attained tertiary level education. Over 45% of the population is educated up to the secondary level and slightly less than a quarter had no formal education. About 13% of the population reported at least an illness/injury in the past one month; most report flu or acute respiratory tract infection. Only a small population (<0.06%) are infected with STDs. Disability was reported by less than 4% of the population with physical disability dominating (Table 1).

**Table 1:** Summary statistics, 2005-2008

**Year of survey**		**2005**	**2006**	**2007**	**2008**
		***Mean***			
**Age**^**a**^		26.71	26.80	26.98	26.67
		(19.37)	(19.45)	(19.51)	(19.19)
		***Proportion *( *% *)**			
**Female**		50.79	50.76	50.75	51.85
**Marital status**	Single/never married	66.15	66.49	66.81	67.69
	Married/living together	27.15	27.23	27.02	26.58
	Widow/widower	4.75	4.51	4.42	4.06
	Divorced/separated	1.94	1.78	1.75	1.67
**Highest education level**	No schooling	23.12	22.89	21.79	21.60
	Primary school	29.53	28.98	28.95	28.52
	Secondary school	45.29	46.06	47.05	47.54
	Tertiary	2.06	2.07	2.21	2.33
**Suffer any illness or injury**		12.63	12.49	11.12	13.75
**Illness type**	Flu	7.20	7.09	5.65	7.66
	Diarrhoea	0.56	0.58	0.42	0.64
	Trauma	0.25	0.37	0.18	0.33
	Tb	0.69	0.59	0.55	0.73
	Drug	0.06	0.05	0.03	0.07
	Depression	0.34	0.36	0.33	0.37
	Diabetes	0.65	0.60	0.55	0.77
	BP	1.53	1.23	1.19	1.55
	HIV	0.15	0.16	0.20	0.28
	STD	0.05	0.04	0.03	0.05
**Suffer any disability**		3.22	3.12	3.01	3.36
**Disability type**	Sight	0.70	0.70	0.63	0.83
	Hearing	0.49	0.50	0.46	0.52
	Speech	0.26	0.21	0.20	0.24
	Physical	1.20	1.08	1.05	1.16
	Intellectual	0.52	0.52	0.52	0.57
	Emotional	0.32	0.27	0.29	0.37
**Number of observations**^**b**^		107,987	105,727	109,975	94,097

Source: Author’s computation based on GHS 2005, 2006, 2007 and 2008.

*Note*: ^a^Standard deviations in brackets; ^b^Total number of individuals in the dataset. Descriptive statistics were computed using only valid responses; so the number of observations will be different for each variable.

**Figure 1:**
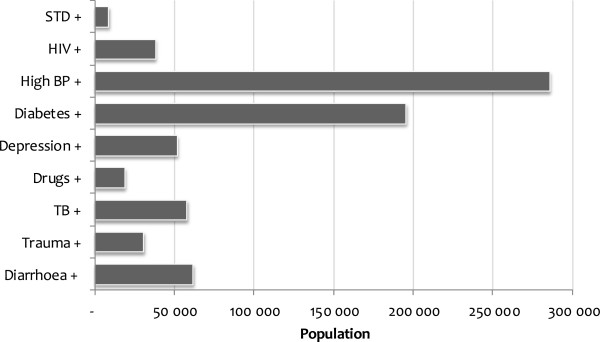
**Population affected by multimorbidity in illness, 2008.***Note*: + indicates the existence of the specified illness in addition to at least one other illness; the numbers have been weighted to represent national figures.

Figure 1 uses the GHS 2008 data to show the number of individuals that are affected by multimorbidity in illness. Each bar indicates the number of people affected by the indicated condition in addition to at least one other illness. In 2008, as shown in Figure 1, multimorbidity in illness that combines BP and at least one other illness is the combination that affects the greatest number of individuals. Over 280,000 South Africans (representing 0.6% of the population) are estimated to report having high BP and at least one other illness. The number is also high for those that indicated a combination of diabetes and other condition(s). Close to 200,000 individuals (representing 0.4% of the population) are estimated to belong to this category. Multimorbidity in illness that combines STDs and other condition(s) has the least reported number of individuals, only about 9,000 (representing 0.02% of the population). Over 50,000 people are estimated to report one of depression, tuberculosis and diarrhoea and at least one other illness.

**Figure 2:**
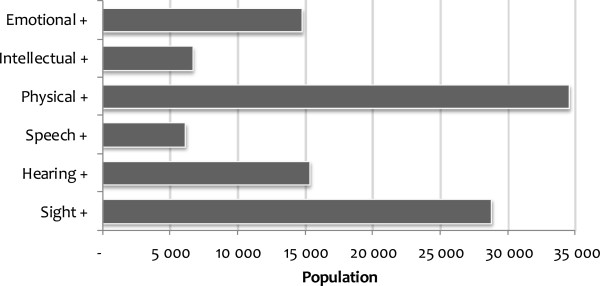
**Population affected by multimorbidity in disability, 2008.***Note*: + indicates the existence of the specified disability in addition to at least one other disability; the numbers have been weighted to represent national figures.

The results for disabilities also show that a substantial number of individuals suffered from multimorbidity in disability in 2008. As shown in Figure 2, about 35,000 people (representing 0.07% of the population) are estimated to have a physical disability in addition to another disability. Data from the same year also show that sight disability (with at least one other disability) was estimated to affect more than 28,000 people in South Africa. Similarly, about 15,000 people, representing 0.03% of South Africans, are estimated to be affected by multiple disabilities combining either emotional disability or hearing disability, with at least one other disability. Speech disability (with at least one other disability) was estimated to affect only about 6,000 individuals.

**Figure 3:**
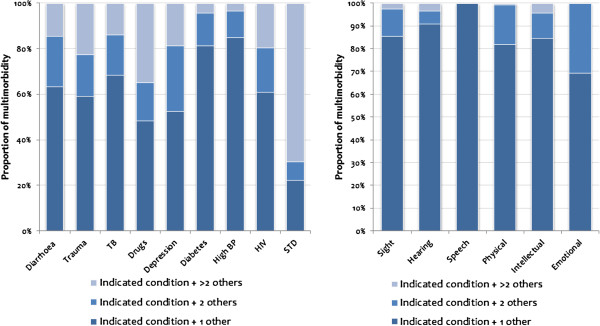
**Distribution of individuals affected by multimorbidity, 2008.***Note*: The numbers have been weighted to represent national figures.

In order to show the intensity of multimorbidity, Figure 3 plots the proportion of individuals with multiple illnesses or disabilities that indicated only two conditions, three conditions and more than three conditions. Generally, as shown in Figure 3, multimorbidity conditions mainly involve only two conditions.

However, this is not the case with STDs and drug abuse. For STDs (drug abuse), 70% (35%) were estimated to report at least three other conditions in addition. These conditions, as shown in Figure 1, affected only a small number of people. For depression, for instance, about 50% reported only one illness in addition, while about 30% reported two other additional conditions. High blood pressure with a substantial number of people, as indicated in Figure 1, was only reported by 15% with at least two additional conditions.

As shown in Figure 3, similar patterns were observed for multimorbidity in disability. Most of the reported multimorbidity in disability occurs as two concurrent conditions. For speech disability, multimorbidity in disability relates to only two concurrent conditions (see also Table 2). For emotional disability, over 30% indicated two additional conditions.

**Table 2:** Average number of conditions suffered by those with multimorbidity, 2008

**Illness**									
	**Diarrhoea**^**+**^	**Trauma**^**+**^	**TB**^**+**^	**Drugs**^**+**^	**Depression**^**+**^	**Diabetes**^**+**^	**High BP**^**+**^	**HIV**^**+**^	**STD**^**+**^
Mean	2.93	3.43	2.91	4.14	3.15	2.36	2.27	3.29	6.42
**Disability**									
	**Sight**^**+**^	**Hearing**^**+**^	**Speech**^**+**^	**Physical**^**+**^	**Intellectual**^**+**^	**Emotional**^**+**^			
Mean	2.18	2.13	2.00	2.19	2.20	2.31			

*Note*: ^+^ indicates the existence of the specified illness or disability in addition to at least one other illness or disability.

A summary of results in Figure 3 is shown in Table 2. Generally, the average number of conditions suffered by those with multimorbidity (illness or disability), with the exception of trauma, drug abuse, depression, HIV, and STDs, is in the neighbourhood of two concurrent conditions.

**Figure 4:**
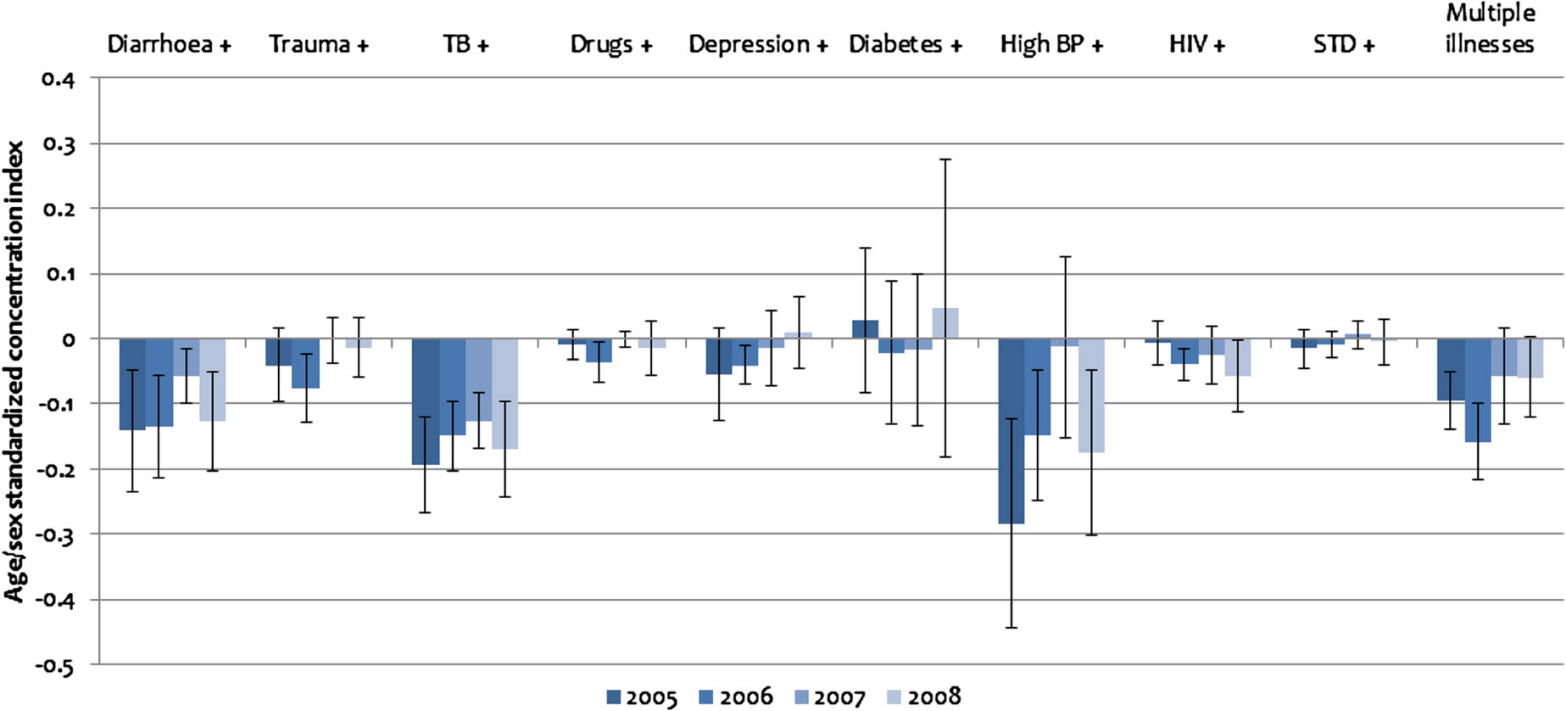
**Standardised concentration indices of multimorbidity in illness, 2005–2008.***Note*: ^+^ indicates the existence of the specified illness in addition to at least one other illness. Standardised concentration indices are based on Erreygers’ normalisation. All estimates are scaled up by 100 to enhance readability. The error bars represent 95% confidence intervals based on robust standard errors.

**Figure 5:**
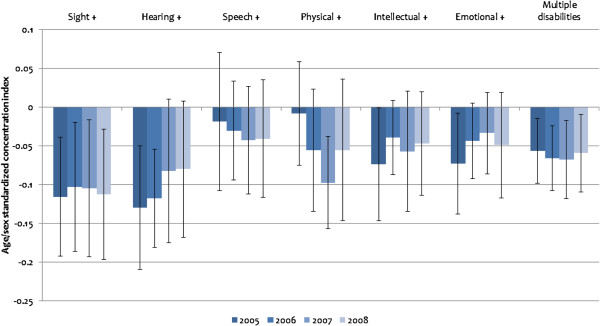
**Standardised concentration indices of multimorbidity in disability, 2005–2008.***Note*: ^+^ indicates the existence of the specified disability in addition to at least one other disability. Standardised concentration indices are based on Erreygers’ normalisation. All estimates are scaled up by 100 to enhance readability. The error bars represent 95% confidence intervals based on robust standard errors.

Figures 4 and 5 show the distribution of multimorbidity by SES using indirectly standardised concentration indices. As indicated earlier, a positive (negative) index signifies a pro-rich (pro-poor) distribution of multimorbidity.

As indicated in Figure 4, between 2005 and 2008, multimorbidity in illnesses is more common among the poor than the rich. Individuals who suffer from diarrhoea and other illnesses are the ones most likely to be among the poorer socioeconomic groups (concentration index = −0.0013 in 2008). The same significant pattern is observed for tuberculosis (concentration index = −0.0017 in 2008) and BP (concentration index = −0.0018 in 2008). The other illnesses are not statistically significant at conventional levels. However, positive indices were sometimes recorded for diabetes and STDs. The last category “multiple illnesses” covers individuals who indicate at least two of the indicated illnesses within the recall period. It assesses multimorbidity in illness irrespective of the combination of illnesses. Apart from 2007, where the indirectly standardised concentration index (−0.0006) is not statistically significant at the 5% level, there is generally a negative and significant concentration of multimorbidity in illness among the poorer socioeconomic groups (concentration index = −0.0009 in 2005, -0.0016 in 2006 and −0.0006 in 2008).

The distribution of multimorbidity in disability in Figure 5 shows a heavy concentration among poorer socioeconomic groups. This relationship is more consistent for disabilities than for multimorbidity in illness. The “multiple disabilities” category in Figure 5, constructed in a similar way as the “multiple illnesses” category in Figure 4, shows a statistically significant greater concentration of multimorbidity in disability among the poor than among the rich between 2005 and 2008 (concentration index = −0.0006 in 2005, -0.0007 in 2006, -0.0007 in 2007 and −0.0006 in 2008).

## Discussion

This paper has shown that multimorbidity affects a substantial number of people in South Africa. It also shows that the poor bear a significantly greater burden of multimorbidity in illness and disability. With reference to the conditions considered in this paper, in 2008 between 0.02% and 0.6% of the population are burdened by multimorbidity in illness while between 0.01% and 0.07% are burdened by multimorbidity in disabilities. With a population of about 48 million people in 2008, this translates into substantial numbers. Most of the multimorbidity here relates to two conditions. However some individuals are affected by three or more disease conditions especially for multimorbidity combining STDs and other conditions. Concentration indices show the extent to which multimorbidity in illness and disability is concentrated among the poor. Though the extent of concentration varies for each multimorbidity condition between the years considered this ‘pro-poor’ distribution is present for both disabilities and illnesses. This conforms to the views expressed by Haveman and Wolfe [28] that in comparison with the nondisabled, “those with disabilities have substantially lower levels of economic well-being in spite of public income support programs” (p.996). These differences in the concentration indices for each multimorbidity condition between the years cannot directly be explained by the analysis. It may not be a reflection of worsening multimorbidity among the rich but differences in the proportion of individuals suffering from these diseases based on the GHS as shown in Table 1.

From the literature, as noted earlier, only a few studies, and limited to high income countries, have explored the relationship between multimorbidity and socioeconomic status [7,9]. There is dearth of information for developing countries. Therefore, it is difficult to compare the results in this paper with any from other developing countries.

In Australia, Walker [9], applying a logistic regression on data from the Australian national surveys, showed that low socioeconomic status significantly increases the probability of having three or more chronic illnesses. In Scotland, Barnet *et al.*[7] found that multimorbidity increases with age, and is correlated with deprivation; where deprivation is an indicator of lower SES. These findings are similar to those obtained in this paper. Multimorbidity in South Africa is significantly associated with lower SES and this is more so for multimorbidity in disability.

The ‘pro-poor’ inequalities in multimorbidity were found generally to decline between 2005 and 2008. This signifies that the burden of multimorbidity is not only concentrated among the poor but also becoming prevalent among the non-poor. These results exhibit a different pattern from those of another study in South Africa that examines inequalities in single illness or disability conditions [4]. That study showed an increasing ‘pro-poor’ distribution for many illnesses and disabilities in South Africa. While it is difficult to explain the differences, it points to differences that may exist in the patterns of inequalities in single disease conditions and multimorbidity over time. Future studies are therefore needed to examine this in other contexts and to investigate the factors that underlie these differences.

Though there is no doubt about the existence of multimorbidity, most “health systems are largely configured for individual diseases rather than multimorbidity” [7] (p.37) thereby making it difficult to manage these conditions simultaneously. To address the issue of multimorbidity, however, requires more than the health system. Based on the Commission on Social Determinants of Health, to tackle the current socioeconomic distribution of multimorbidity in South Africa requires an integrated approach that goes beyond the health sector and which recognises the importance of the social determinants of health [29]. As argued elsewhere in the broad context of health inequality, this “requires a coherent intersectoral approach that will account for the interrelatedness of factors that are associated with health inequalities in South Africa” [30] (p.762). This strategy must recognise that the burden of diseases, including that of multimorbidity, is greater among the poor than among the rich.

This study has some limitations. The GHS data sets do not include all the likely disease conditions, e.g., cancers. However, the conditions included in the GHS account for most of the premature mortality, measured by years of life lost, in South Africa [4]. Further, the data were collected from general households and exclude institutions such as nursing homes and hospices that house people who are very likely to suffer multimorbidity. The data are self-reported measures of illnesses and disabilities but as argued elsewhere [4], the presence of most of these illnesses can only be known through medical diagnosis while, in the case of disabilities, they are more likely to be self-diagnosed or easily observed. While it is difficult to postulate about the nature of inequality, the burden and extent of multimorbidity in South Africa may thus be underestimated.

## Conclusion

Multimorbidity affects a substantial number of South Africans. Its distribution shows that the poor bear a greater burden. This is more so for disability than for illness. The dearth of information on this distribution in many developed and developing countries can be attributed to the lack of data that link socioeconomic status with the incidence of multimorbidity. Based on the findings in this paper, there is a need to design policies that address multimorbidity in South Africa. These policies, with insights from the World Health Organisation’s Commission on Social Determinants of Health, should be coherent and involve more than the health sector. Further, there is a need to design national surveys that specifically assess multimorbidity.

## Competing interests

The author declares that there are no competing interests.

## Supplementary Material

Additional file 1 Questions relating to illnesses and disabilities in the General Household Surveys. Click here for file
