# Supplementary material for: Inequalities in multimorbidity in South Africa
Source: Int J Equity Health. 2013 Aug 20;12:64. doi: 10.1186/1475-9276-12-64 (PMC3765402; doi:10.1186/1475-9276-12-64)
Supplement: Additional file 1 — Questions relating to illnesses and disabilities in the General Household Surveys. [file 1475-9276-12-64-S1.docx]

Appendix 1: Questions relating to illnesses in the GHS

**Ask for everyone in the household**

Read out: Now I am going to ask you health-related questions for each member of the household

1.19 During the past month, did …… suffer from any of illnesses or injuries? [Yes/No] ( 🡪 if No, skip the next question)

1.20 What sort of illnesses or injuries did …… suffer from?

Did …… suffer from ……

01 = Flu or acute respiratory tract infection

02 = Diarrhoea

03 = Sever trauma (e.g. due to violence, motor vehicle accident, gunshot, assault, beating)

04 = Tuberculosis (TB) or severe cough with blood

05 = Abuse of alcohol or drugs

06 = Depression or mental illness

07 = Diabetes

08 = High or low blood pressure

09 = HIV/AIDS

10 = Other sexually transmitted disease

11 = Other illness or injury

Appendix 2: Questions relating to disabilities in the GHS

**Ask for everyone in the household**

Read out: I am going to ask about disabilities experienced by any persons within the household

1.28 Is …… limited in his/her daily activities, at home, at work or at school, because of a long-term physical, sensory, hearing, intellectual, or psychological condition, lasting six months or more? [Yes/No] ( 🡪 if No, skip the next question)

1.20 What difficulty or difficulties does …… have?

Is it ……

1 = Sight (blind/severe visual limitation)

2 = Hearing (deaf, profoundly hard of hearing)

3 = Communicating (speech impairment)

4 = Physical (e.g. needs wheel chair, crutches or prosthesis; limb or hand usage limitation)

5 = Intellectual (serious difficulties in learning, mental retardation)

6 = Emotional (behavioural, psychological problems)

7 = other, specify in the box at the bottom
